# Draft Genome Sequence of Saccharomyces cerevisiae Strain Hm-1, Isolated from Cotton Rosemallow

**DOI:** 10.1128/MRA.01184-18

**Published:** 2018-10-04

**Authors:** Hiro Takahashi, Erika Sakagawa, Ikuya Sakakibara, Chiyoko Machida, Shido Miyaki, Anna Takahashi, Shinnosuke Onai, Shuichi Fukuyoshi, Akinori Ohta, Kenji Satou, Shin Kanamasa

**Affiliations:** a Graduate School of Medical Sciences, Kanazawa University, Kakuma-machi, Kanazawa, Japan; b Graduate School of Horticulture, Chiba University, Matsudo, Chiba, Japan; c Fundamental Innovative Oncology Core Center, National Cancer Center, Chuo-ku, Tokyo, Japan; d Graduate School of Bioscience and Biotechnology, Chubu University, Kasugai, Aichi, Japan; e College of Bioscience and Biotechnology, Chubu University, Kasugai, Aichi, Japan; f Faculty of Information Technologies and Control, Belarusian State University of Informatics and Radio Electronics, Minsk, Belarus; g Institute for Theoretical Physics, Kanazawa University, Kakuma-machi, Kanazawa, Japan; h Institute of Medical, Pharmaceutical and Health Sciences, Kanazawa University, Kakuma-machi, Kanazawa, Japan; i Faculty of Biological Science and Technology, Institute of Science and Engineering, Kanazawa University, Kakuma-machi, Kanazawa, Japan; University of Arizona

## Abstract

Saccharomyces cerevisiae strain Hm-1 is a yeast isolated from the flower of cotton rosemallow. This yeast is used for the production of Seishu, a traditional Japanese refined sake. Here, we report the strain’s draft genome sequence. With this genomic information, the brewing characteristics of the strain can be better understood.

## Abstract (precis)

Saccharomyces cerevisiae strain Hm-1 is a yeast isolated from the flower of cotton rosemallow. This yeast is used for the production of Seishu, a traditional Japanese refined sake.

## ANNOUNCEMENT

Seishu (refined sake) is a traditional Japanese liquor made from rice. One of the characteristics of its manufacturing method is that it is brewed by parallel double fermentation via saccharification and alcohol fermentation by simultaneous use of the fungus Aspergillus oryzae and the yeast Saccharomyces cerevisiae. Thus, the refined sake has a high alcohol content compared to that of other brewed alcoholic beverages, such as wine. Like other brews, the role of yeast in the brewing of refined sake is important. Yeast produce metabolites, such as ethanol and various organic acids, while also taking up saccharides and some of the organic acids contained in the raw materials, and these metabolites become important components that determine the taste and aroma of the refined sake. We aimed to develop a sake of unique character; we searched for new yeasts from various kinds of plants, and a yeast suitable for the brewing of refined sake was isolated from the flower of cotton rosemallow (Hibiscus mutabilis). A brewing test of sake produced from the yeast S. cerevisiae strain Hm-1 was conducted. The strain showed differences in its ability to produce alcohol, organic acid, and aromatic components and in its ability to take up amino acids compared with those of conventional yeast (1). To make better refined sake, as well as to understand the differences between these phenotypes at the molecular level, we analyzed the genome sequence of the yeast strain Hm-1 in this study.

The genomic DNA of strain Hm-1 was isolated from a liquid-submerged culture grown in yeast extract-peptone-dextrose (YEPD) medium at 30°C. Sequencing by the TruSeq DNA library (paired-end 2 × 300-bp reads) generated 4,535,936 reads. Removal of the sequencing primers and trimming of the low-quality read regions from the obtained short reads were conducted using CLC Genomics Workbench version 11.0.1 (Qiagen, Valencia, CA, USA) with the default parameters. *De novo* assembly was also conducted using CLC Genomics Workbench. Contigs shorter than 200 bp were discarded. The resulting genome assembly had a length of 11,794,612 bp, divided into 460 contigs. The *N*_50_ contig length was 258,733 bp, the GC content was 38.1%, and the genome coverage was 108.0×. Prediction of the coding regions of chromosomes was performed using AUGUSTUS (2) and implemented in Blast2GO version 5.1.13 (Qiagen), with S. cerevisiae S288C as a gene model. The estimated number of genes in the draft genome was 5,426. This genomic information can provide insight into the genetic basis for the brewing characteristics of this strain.

### Data availability.

The draft genome sequence for S. cerevisiae Hm-1 has been deposited in DDBJ/ENA/GenBank under accession numbers BHFU01000001 to BHFU01000460. The SRA/DRA/ERA accession number is DRA007299.
